# Perceived impacts of North Americas first de-medicalized safer supply program

**DOI:** 10.1186/s13011-025-00642-0

**Published:** 2025-03-10

**Authors:** Jeremy Kalicum, Eris Nyx, Mary Clare Kennedy, Thomas Kerr

**Affiliations:** 1 Drug User Liberation Front, Vancouver, Canada; 2 Department of Medicine, University of British Columbia, St. Paul’s Hospital, 608-1081 Burrard Street, Vancouver, BC V6Z 1Y6 Canada; 3 British Columbia Centre on Substance Use, 400-1045 Howe Street, Vancouver, BC V6Z 2A9 Canada; 4 University of British Columbia - Okanagan School of Social Work, Vancouver, BC Canada

**Keywords:** Compassion club, Safer supply, Overdose, Harm reduction, Drug user liberation front

## Abstract

**Background:**

The Drug User Liberation Front led an evaluation of a non-medicalized model of safer supply known as a “Compassion Club.” This club sourced, rigorously tested, packaged, and accurately labeled certain illicit substances and then provided them to club members at cost in order to investigate the effects and feasibility of a non-medical model of safer supply. Operating for 14 months, the club provided low-cost, quality-controlled illicit substances to individuals at risk of fatal overdose in Vancouver, Canada’s Downtown Eastside neighbourhood. This study was undertaken to explore perceived impacts of the Compassion Club among its participants, as well as their perceptions regarding how the Club could be improved.

**Methods:**

At the one-year time point of club operations 43 participants from the club’s membership completed an interviewer-administered survey which is utilized in this cross sectional analysis. Descriptive statistics were employed to assess the perceived influence of club membership on various factors, including drug use patterns, harm reduction practices, financial outcomes, housing stability, and overall well-being.

**Results:**

Applicable responses reported benefits from membership in the Compassion Club, including reduced drug use (64.3%), decreased reliance on illicit markets (86.7%), lowered risk of overdose (90.0%), and increased likelihood of using sterile equipment (84.6%). Mental health (74.2%), physical health (63.3%), and overall well-being (70.0%) were also noted improvements. Suggestions for club improvement included extended operating hours, broader substance selection, and improved accessibility.

**Conclusion:**

The reported reductions in drug use and improved adherence to harm reduction practices described herein underscore the perceived benefits of this unique program. Compassion Clubs represent a distinct strategy to mitigate overdose risk and enhance the well-being of drug users. These insights advance ongoing dialogues on overdose prevention strategies, urging further research to refine non-medicalized approaches within the evolving landscape of interventions.

## Introduction

Since the declaration of a public health emergency in response to the overdose crisis in British Columbia (BC), Canada in 2016, the situation has progressively deteriorated. A total of 2558 overdose deaths due to the unregulated drug supply were recorded in BC in 2023, which equates 46.3 unregulated drug deaths per 100,000 population—an unprecedented figure in the history of overdose deaths in the province [1]. In effort to respond to overdose and its related harms, there has been growing support to separate people from the unregulated illicit drug market as a strategy to mitigate accidental drug overdoses [2–4]. Safer supply, or regulated, quality-controlled alternatives to the unregulated illicit drug market, has been a focus of this approach, reasoning that, in comparison to the unregulated illicit drug market, access to substances with known potency and composition can reduce risk of overdose [2, 5]. Recently, a recommendation from BC’s Chief Coroner called for the implementation of non-prescriber-based models of safer supply to reduce overdose deaths [6].

Efforts to offer alternatives to the unregulated illicit drug supply have garnered attention, with a predominant focus on medicalized interventions providing prescription alternatives within highly controlled settings [7, 8]. Benefits of these programs have included reduced use of illicit cocaine by participants of a prescribed psychostimulant program [9], and reductions in mortality for a heroin prescription program [10]. Further, a recent scoping review revealed that engagement in prescribed safer supply programs is connected with better mental health, increased access to healthcare, decreased involvement in criminal activities related to obtaining drugs, lower frequency of drug use, and fewer instances of opioid toxicity events [11]. While such initiatives do appear to have relatively high public support, government adoption into broad policy change remains a challenge [12], and prescriber uptake of these initiatives remains difficult [13].

Pioneering a departure from this medicalized approach, the Drug User Liberation Front (DULF), a non-profit organization, implemented a non-medicalized model of market regulation via a Compassion Club, marking the first known occurrence of its kind [14]. Over a 14-month period, DULF operated the compassion club, a non-profit initiative providing at-cost, laboratory-tested, and accurately labeled cocaine, heroin, and methamphetamine to individuals already engaged in substance use, at risk of overdose, and residing in Vancouver’s Downtown Eastside. Although requested, fentanyl and crack cocaine were not available from the compassion club due operational capacity and concerns of being able to reliably source a reliable, consistent supply of these substances. Participants were allowed to purchase cocaine, methamphetamine, and heroin in quantities of up to 14 g per week. The club, operational up to four days a week at a fixed physical location, also offered on-site overdose prevention and distributed harm reduction supplies [15].

There is a notable gap in the existing literature concerning research on non-medicalized alternatives to the unregulated drug market. To address this gap, the compassion club project underwent a rigorous and ongoing evaluation, which included inquiries into participants’ perceptions of the club’s direct impact on their lives and health. This paper presents findings on these perceived impacts of the DULF Compassion Club on its participants, as well as their perceptions regarding how the club could be improved.

## Methods

### Study setting and design

Data for this study were obtained from interviews completed by an open prospective cohort of 43 DULF Compassion Club members. As described previously (Kalicum et al., 2024), this longitudinal evaluation was accomplished via interviewer-administered surveys conducted upon baseline entry, every subsequent three months, and at the end of the program. The surveys were adapted from other surveys used by studies in Vancouver’s Downtown Eastside, including the Safer Alternative for Emergency Response (SAFER) Evaluation [16] and Vancouver Injection Drug User Survey (VIDUS) [17]. Prior to administration, the surveys underwent approval by a community-based ethics review, DULF’s indigenous advisory board, the DULF Board of Directors, and the Vancouver Area Network of Drug Users. Ethical approval for secondary analysis of the data was obtained from the University of British Columbia’s Behavioural Research Ethics Board (H23-02497).

Trained research assistants obtained informed consent and administered the surveys, each lasting approximately two hours. All members of the compassion club were extended invitations to partake in the evaluation, with a $50 stipend offered at each study visit. The present analysis was restricted to a cross-sectional analysis of survey data collected at the one-year time point of the program operating, which includes 17 people from cohort one with one year in the compassion club, 19 people from cohort two with nine months in the club, and 7 people from cohort three with four months in the club. By using the one-year dataset for the analysis, we aimed to capture a representative snapshot of the participants’ experiences.

### Cohort description and recruitment

Participants in the DULF Compassion Club were selected via a lottery from a pool of self-referred applicants comprising members from various drug user groups in Vancouver, including the Western Aboriginal Harm Reduction Society, BC Association of People on Opiate Maintenance, The Coalition of Peers Dismantling the Drug War, The Tenant Overdose Response Organizers, and the Vancouver Area Network of Drug Users. To be eligible for this lottery, potential club participants had to meet the following criteria: be over the age of 19, currently be at risk of overdose (including all active users of unregulated illicit drugs), use substances the club would provide (such as cocaine, methamphetamine, and heroin), and be a member of one of the previously mentioned drug user groups in Vancouver. To ensure community knowledge of the program, a DULF founder went to the weekly open membership meetings of all groups to gather names of eligible people for one month. The names of eligible and interested members of the groups were all then written on separate pieces of paper, placed in a container and participants were randomly selected from this container. If the potential participant was still interested in joining the compassion club they would be admitted to the study, if not another name would be drawn from the container. Enrollment into the club was increased overtime as the capacity of the club increased.

### Primary outcomes

Descriptive statistics, in the form of percentages, were tabulated for each perceived impact. Surveys at each time point included questions pertaining to participants’ perceived impacts of the compassion club. Questions all began with “Being a member of the DULF Compassion Club…” and varied with the following additions: helped me to reduce my drug use; helped me stop using drugs; reduced my cravings/withdrawal; helped reduce my use of the illicit market; reduced my overdose risk; made me more likely to use clean/sterile drug use equipment; made me more likely to use drugs slowly and/or taste drugs first; made me more likely to carry Naloxone; made me more likely to have my street drugs checked; helped reduce my reliance on illegal activities; improved my income; improved my housing stability; made me less likely to experience of physical assault/violence; made me less likely to have contact with police; helped me to improve my connections with friends and family; increased my use of other health or social services; improved my pain management; improved my physical health; improved my mental health; and improved my overall health. Participants responded on a Likert scale consisting of the following response categories: Strongly agree, agree, neutral, disagree, strongly disagree, and not applicable. Responses we grouped as strongly agree/agree vs. neutral vs. disagree/strongly disagree. Not applicable responses were interpreted as the participant not perceiving the program as relevant to the outcome being queried. For instance, when asked whether the program had helped reduce their reliance on illegal activity or made them less likely to experience physical assault/violence, a “not applicable” response might include statements such as, “I was never reliant on illegal activity,” or “I was never at risk of physical assault.” Not applicable responses were excluded from the measurement and all others being subsequently referred to as “applicable responses.”

Participants were also asked if they believed the club could by improved via a binary yes vs. no response. Those who reported “yes” were subsequently queried about how the club could be improved using a list of items read aloud by the interviewer and asked to select all that apply. These items included longer operating hours; shorter wait time; other drug options, please specify; if there were changes to program rules/regulations, please specify; if staff treated me better; if the physical space was improved, please specify; and other, please specify. Responses were analyzed using descriptive statistics.

## Results

Initial membership in the compassion club consisted of 21 participants. However, as the club’s capacity expanded another 28 participants were admitted, ultimately cumulating in 49 participants in the compassion club and the connected study. Three of these participants were lost to follow-up, two withdrew from the study, and one died prior to accessing the club, leaving 43 participant respondents for this analysis at the one-year timepoint. Among these participants, 11 (25.6%) identified as women, 26 (60.5%) as men, and 6 (14.0%) as non-binary. In terms of ethnicity/ancestry, 24 (55.8%) identified as being white, 20 (46.5%) as Indigenous, and 4 (9.3%) as other people of colour.

**Fig. 1:**
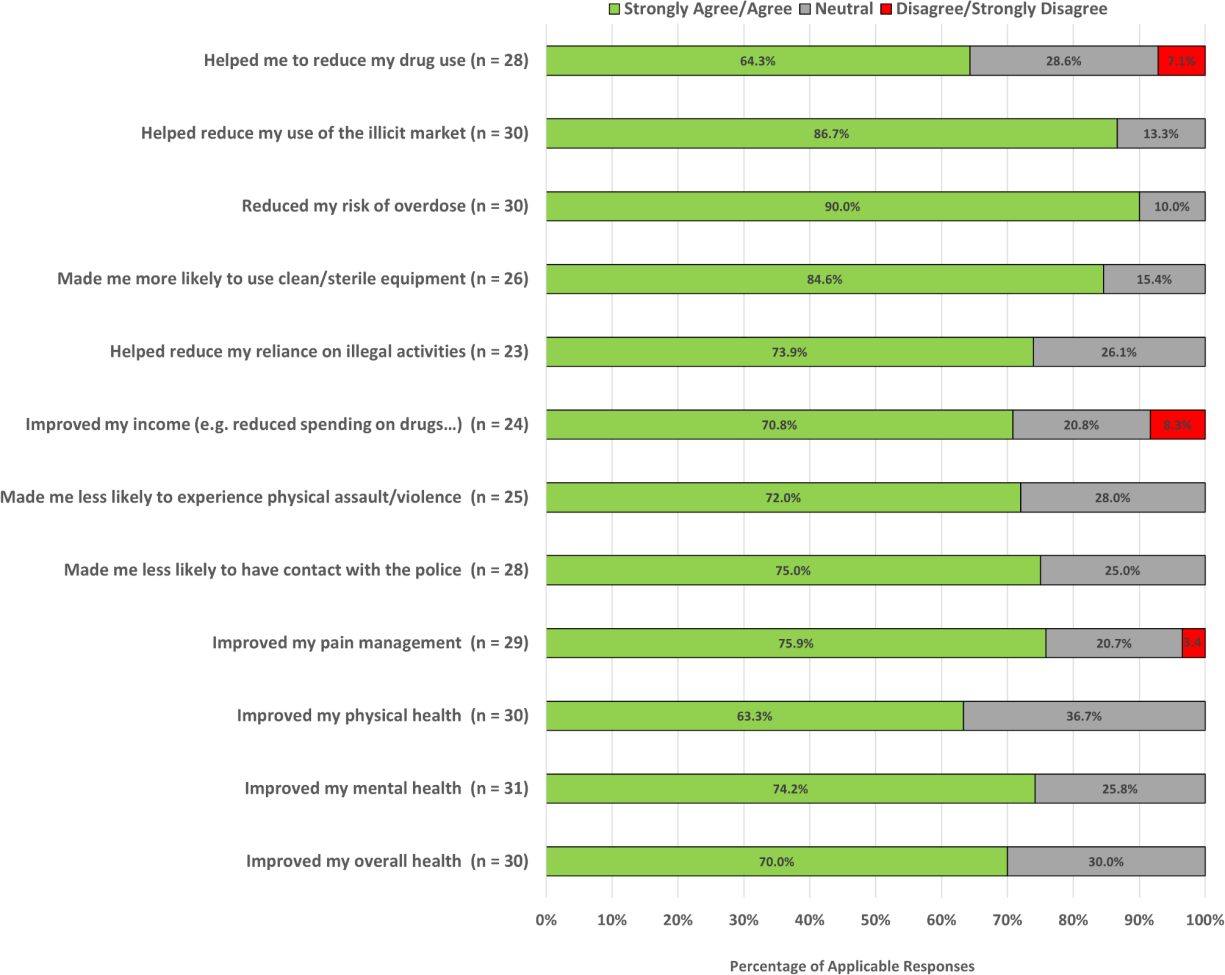
Queried Responses to Questions Regarding the Impact of Participation in the DULF Compassion Club (*n* = 43)

A substantial portion of applicable responses reported positive impacts on their drug use patterns. Specifically, 18 (64.3%) of the 28 applicable responses indicated that being a member of the compassion club helped them reduce their drug use, and 27 (90.0%) of 30 perceived a reduction in the risk of overdose. Addressing engagement with the illicit drug market, a substantial majority of participants experienced positive changes attributed to compassion club membership. Notably, 26 of 30 (86.7%) applicable responses reported a decrease in use of the illicit market. Positive impacts on participants behaviours were also reported, with 22 (84.6%) of 26 applicable responses reporting an increased likelihood of using clean and sterile equipment. The club’s impact on social connections, mental health, and overall well-being was also assessed. In total, among applicable responses 21 (70.0%) of 30 indicated that their overall health had improved. Additionally, 17 (73.9%) of 23 reported a reduced reliance on illegal activity, and 18 (72.0%) of 25 perceived a reduction in the likelihood of experiencing physical assault or violence, and 21 (75.0%) of 28 reported being less likely to have contact with the police. Additional details and responses are presented in Fig. 1.

Notably, not applicable responses in the responses to club impact on participants among the presented results ranged from 12 to 20 “not applicable” responses. Further, none of the perceived compassion club impact questions were skipped by study participants. Non applicable responses counts are 15 for reduced drug use, 13 for illicit market use, 13 for overdose risk, 17 for use of sterile equipment, 20 for illegal activity, 19 for income, 18 for physical assault/violence, 15 for police contact, 14 for pain management, 13 for physical health, and 14 for overall health. Due to the high non-applicable response rate, the following questions related to impacts of the compassion club were excluded from the analysis: helped me stop using drugs, reduced my cravings/withdrawal, made me more likely to use drugs slowly and/or taste drugs first, made me more likely to carry Naloxone, made me more likely to have my street drugs checked, improved my housing stability, helped me to improve my connections with friends and family, and increased my use of other health or social services.

When prompted on ways that the club could be improved, 37 (86.0%) of the 43 participants expressed the club could be improved and subsequently identified several areas for improvement. Among those who believed the club could be improved, 33 (89.2%) requested longer operating hours, 25 (67.6%) requested additional substances be available for purchase with 14 (37.8%) requesting fentanyl, 14 (37.8%) requesting crack cocaine, 3 (8.1%) requesting benzodiazepines, 3 (8.1%) requesting ketamine, 3 (8.1%) requesting other psychedelics and single requests for Percocet, M-Eslon, cannabis and MDA, also known as 3,4-Methylenedioxyamphetamine, availability. Finally, 8 (21.6%) of the 37 believed the physical space of the compassion club could be improved and 3 (7.0%) believed the club should be located in a different area.

## Discussion

In this study involving 43 individuals enrolled in the DULF Compassion Club, findings suggest that participation in this program was associated with various self-reported positive outcomes. Of note, 90.0% of applicable responses indicated a reduction in overdose risk, and 64.3% reported the club helped them reduce their drug use. The results further suggest a range of additional positive impacts of the DULF Compassion Club on participants’ health and well-being, including increased safer drug use and harm reduction practices, economic stability, and fewer interactions with law enforcement. The most common suggestions for improving the club reported by participants were the need to increase the accessibility of the club via extended hours of operation and to provide additional substances, in particular fentanyl and crack cocaine.

The participant-reported impacts of the DULF Compassion Club observed in the present study appear to be among the first in the emerging research literature on non-medicalized safer drug supply. These findings add to a growing body of research documenting beneficial outcomes of safer supply programs [11]. Indeed, previous studies of such programs have reported a range of positive impacts including reductions frequency of unregulated drug use and overdose risk [14, 18–20], reductions in emergency department visits and hospital admissions [21], reduced exposure to violence associated with drug procurement [22], improved pain management [8], reductions in criminal activity [8, 19, 23], improved health and well-being [8], reduced interactions with law enforcement [19], and improvements in overall health [8, 19, 22]. However, most of these studies, with the exception one related to a complementary component of this research [14], have focused on medicalized models of safer supply, primarily those that prescribe tablet hydromorphone. The present study builds upon this emerging body of research by shedding light on the impacts of a non-medicalized safer supply program on various self-reported health and wellbeing indicators, a crucial aspect of the harm reduction continuum of care. The positive outcomes observed in our study resonate with the longstanding demand from people who use drugs and other experts for a stable, predictable, and easily accessible drug supply to prevent overdoses amid the ongoing toxic drug crisis, including through non-medical safer supply models [4, 24, 25].

The findings reported herein also build upon research documenting the protective effects of “trusted dealers,” where drug dealers were trusted by their clients to employ consumer protection and quality assurance measures, and clients characterized these relationships as reducing substance use-related harms, including overdose [26]. “Trusted dealers” are thus one of many methods that can be employed to increase the reliability of drugs that people consume and resultingly reduce substance related harms. In a similar manner to such “trusted dealers,” the DULF Compassion Club acted to implement controls in a more regulated and enhanced manner with additional oversight and accountability processes. However, points of contention remain between these two concepts related to the level of regulation and oversight needed to maximize benefits while minimizing harms. As such, further research should be done to comprehensively compare the impacts of differing degrees of regulation on safer supply programs.

Despite the perceived positive impacts of the DULF Compassion Club observed in the present study, it is notable that the majority of participants reported that the program could be improved by expanding operating hours and providing additional drug options, including fentanyl and crack cocaine. These programmatic issues have similarly been identified as barriers to engagement among clients of medicalized safer supply programs [27]. These findings suggest that participants may further benefit from safer supply programs that are appropriately resourced to reduce these barriers and provide a wider variety of substances, including more potent options. These findings also point to the need for additional research to better understand how to optimize the effectiveness of de-medicalized safer supply interventions.

This study has limitations that should be noted. First, the absence of a control group to further establish validity of the results is a limitation. Nonetheless, considering the descriptive nature of this analysis, a control group is not necessary to reflect participant perceived impacts. Second, a small sample size and unique environment may limit generalization of these findings beyond this distinct population and setting. However, the purpose of this study was to capture and characterize the perceptions of DULF club members specifically. Next, potential biases introduced by self-reporting, including socially desirable responding and recall bias, may have affected our results. However, past reviews reported validity of self-reporting from people who use drugs [28, 29]. Further, the variability in exposure among participants may also leave the study vulnerable to temporal bias as differences in duration in club participation may confound results. The variability in exposure among participants, though a limitation, was an unavoidable aspect of the recruitment process, allowing for the inclusion of a diverse sample that better represents the range of real-world participation and engagement with the program. There is also potential for selective attrition bias as participants who withdrew or were lost to follow up did not provide data for the analysis and may be more likely to have negative perceptions of the club. However, two participants withdrew from the study and their withdrawal did not occur as a result of dissatisfaction with the program, and three participants were lost to follow up, but substantial efforts were made to contact all participants regardless of their level of engagement with the club. Nevertheless, the low response rate to some survey items introduces the potential for non-response bias, which could confound the findings.

The aforementioned limitations underscore the need for further research employing robust methodologies and comparative analyses to build upon and confirm these findings. Indeed, a comparative assessment with medicalized interventions could elucidate the specific strengths and limitations of non-medicalized harm reduction models, addressing the multifaceted challenges posed by substance use and overdose. Nevertheless, and despite its limitations, this study has implications for research and policy development specific to safer supply and overdose prevention.

## Conclusion

In summary, this evaluation focused on self-reported impacts of the DULF Compassion Club and observed a range of positive impacts for participants, including reductions in drug use, improvements in harm reduction practices, financial benefits, and positive effects on mental and overall health. While these findings contribute to the existing literature on safer supply programs, there remains a need for further research to rigorously evaluate the impacts of non-medicalized safer supply programming. Further, examining the comparative effectiveness of non-medicalized models against established interventions remains a crucial area for exploration in advancing harm reduction strategies and optimizing support for individuals at risk of overdose.

## Data Availability

The authors confirm that the data supporting the findings of this study are available within the article.

## Declarations

***CRediT Authorship and Contribution Statements***.

**Jeremy Kalicum**: Study Design - Writing – review & editing, Writing – original draft, Conceptualization, Data analysis. **Eris Nyx**: Study Design - Writing reviewing & editing, Conceptualization. **Thomas Kerr**: Study Design - Writing – reviewing and editing. Supervision. Methodology, Conceptualization. **Mary Clare Kennedy**: Writing – review & editing,

## Abbreviations

BC: British Columbia
DULF: Drug User Liberation Front
MDA − 3,4: Methylenedioxyamphetamine
SAFER: Safe Alternative for Emergency Response
VIDUS: Vancouver Injection Drug User Study
